# Evaluation of antimicrobial effect of MTA, calcium hydroxide, and CEM cement

**Published:** 2007-10-02

**Authors:** Saeed Asgary, Farshid Akbari Kamrani, Soudabeh Taheri

**Affiliations:** 1 Department of Endodontics, Iranian Center for Endodontic Research, Dental Research Center, Shahid Beheshti University MC, Tehran, Iran.; 2 Researcher of Dental Research Center, Shahid Beheshti University MC, Tehran, Iran.; 3 Instructor of Microbiology Dept., Medical School, Shahid Beheshti University MC, Tehran, Iran.

**Keywords:** Antibacterial agent, Calcium hydroxide, Dental material, Endodontics, Mineral Trioxide Aggregate

## Abstract

**Introduction:** The antibacterial effects of calcium hydroxide (CH), mineral trioxide aggregate (MTA), and calcium enriched mixture (CEM) cement against various spp. of microorganisms were assessed *in-vitro* using agar diffusion test.

**Materials and Methods:** A base layer of Petri plates was made using Muller-Hinton agar. Four cavities were made in agar and filled with freshly mixed materials after 24 h. The microorganisms were seeded by pour plate. The plates were pre-incubated for 2 h at room temperature followed by incubation at 37˚C for 24 h. The inhibition zones diameters were measured by an independent observer.

**Results:** The highest mean diameters of growth inhibition zones were founded around CH and CEM cement. According to One-way ANOVA there was a significant difference among test groups (p<0.001); Post Hoc test revealed no significant difference between the mean zones diameter of CH and CEM cement. However, there was significant difference among CH and CEM cement in comparison with MTA group (p<0.001).

**Conclusion:** It appears that CEM cement is a potent antibacterial agent like CH.

## INTRODUCTION

Microorganisms are the main etiologic factors for the development of pulp and periapical inflammation (1). 

Elimination of microorganisms during root canal treatment by instrumentation, irrigation and intracanal medication has been always an important part of successful endodontic treatments (2). Although superficial adhering of microorganisms to root canal dentin might be expected to be killed easier than those protected in depths of dentinal tubules, but microorganisms inside the dentinal tubules might also be challenged by antimicrobial components leaching from the irrigation solution, intracanal medication, and endodontic filling and sealing materials (3). However, even after these chemomechanical procedures, bacteria might still be found inside the dentinal tubules with the potential for disease to persist or emerge (4).

Endodontic treatment outcome will depend on the effective seal to prevent future recontamina-tion as well as successful reduction or elimination of the associated microorganisms. Since many existing biomaterials may not provide a perfect seal, it was expected that these materials also prevent bacterial growth (5). Therefore, antimicrobial testing of biomaterials should consider this effect. The agar-diffusion test (ADT) is the most commonly used technique for evaluating this property of dental materials (6).

Numerous studies have been performed to assess the antibacterial activity of different materials used in endodontic treatment. Calcium hydroxide (CH) was first introduced to dentistry in 1920 (7). Some biological properties attributed to CH such as induction of hard tissue formation (8), inhibition of root resorption (9), antibacterial action (10), and tissue dissolution (11). Because of these biological properties, CH has been recommended for many clinical usages in endodontics.

Mineral trioxide aggregate (MTA), which was introduced in 1993 (12), have been examined since 1995 as potential antibacterial material (5). MTA is a powder consists of fine hydrophilic particles that in the presence of water forms a colloidal gel that solidifies to form hard cement within approximately 4 h (13). ProRoot MTA is marketed in gray-colored (GMTA) and white-colored (WMTA) preparations. GMTA may cause tooth discoloration particularly when it is used to cap or seal a perforation, where aesthetics is the priority. WMTA has been introduced in order to address this issue (14). Major compositional differences in the concentrations of carborundum (Al_2_O_3_), periclase (MgO) and especially FeO between GMTA and WMTA were reported. The values of these elements were found to be considerably lower in WMTA (15). According to recent studies, MTA is a biocompatible dental material and it was suggested that this biological properties may be due to its excellent sealing ability (16), high alkalinity (17) induction of hard tissue formation (18), and antibacterial effects (5). Because of its physical and chemical properties, the use of MTA as a biomaterial for a wide variety of endodontic treatments has been recommended (19).

Although MTA has excellent biocompatibility, it has a delayed setting time (13), poor handling characteristics (20), while it is an expensive material. 

CEM cement which was introduced in 2006 (21) was formulated using different calcium compounds. The results of our other studies (unpublished data) revealed that the mixed NEC comprises water-soluble calcium and phosphate, and immediately forms hydroxy-apatite during and after setting time. This cement is biocompatible (22) and forms an effective seal when used as root-end filling material and the results is comparable with different root-end filling materials (21,23).

The aim of the present *in vitro* study was to compare the antimicrobial activities of CH, MTA, and CEM cement on four microorganisms commonly associated with endodontic infections using the agar-diffusion test.

## Materials and Methods

In this study, we had three experimental groups as follows: group 1- calcium hydroxide (Kerr, Orange, CA, USA ); group 2- ProRoot MTA tooth-coloured formula, (Dentsply Tulsa Dental, Tulsa, Oklahoma, USA); and group 3- calcium enriched mixture (CEM) cement. 

The study was conducted on double-layered plates, in which the base layer was made of 10 mL of sterilized Muller-Hinton agar (MH) poured in 2x10 cm sterilized Petri plates. Four uniform cavities (4 mm diameter, one for each test material) were punched at equidistant points in agar by means of a sterile copper coil after 24 hours. The cavities were filled immediately by materials after being mixed according to the manufacturer’s instructions.

Antibacterial activities of the selected materials were evaluated against the *Pseudomonas aeruginosa*, *Enterococcus faecalis*, *Staphylococcus aureus*, and* Escherichia coli* using an agar diffusion method. The strains were obtained from the Department of Microbiology, Faculty of Medicine of SBMU. After activation from stock culture, microorganisms were maintained in MH broth until used. Overnight cultures of the microorganisms were used. All the microbial strains were grown at 37˚C for 24 h in MH broth and then seeded into 15mL of the MH agar, to produce a turbidity of 0.5 on the Mc Farland scale, which corresponds to a concentration of 10^8^ colony forming unit mL^-1^. This broth was used as the second layer. The seeded agar was added over the plates immediately after the insertion of freshly mixed test materials. The plates were kept at room temperature for 2 h for pre-diffusion of the materials and then incubated at 37˚C for 24 h. 

**Table-1:** The antibacterial activity of test materials toward four bacterial spp. The zones of growth inhibition presented in millimeters.

Materials	Microorganisms			
	***E. coli***	***S. aureus***	***P. aeruginosa***	***E. faecalis***
**CH**	**5.125(0.353)** *	**6.500(0.462)**	**3.812(0.372)**	**6.812(0.258)**
**MTA**	**2.937(1.208)**	**4.375(0.443)**	**2.250(0.462)**	**5.187(0.458)**
**CEM**	**5.375(0.64)**	**6.375(0.79)**	**3.375(0.353)**	**7.062(0.176)**

* Mean (standard deviation)

A total number of 26 plates were employed; the plates were divided randomly into three test groups having eight plates each so micro-organisms were tested eight times.

Positive and negative controls were prepared, maintaining the plates with and without inoculums, for the same period and under identical incubation conditions. All assays were carried out under aseptic conditions. 

The diameter of bacterial growth inhibition zones was measured with a millimeter ruler with accuracy of 0.5 mm in two perpendicular locations for each sample by an independent observer.

Statistical analysis was performed using a one-way ANOVA for the mean zones of growth inhibition among the materials tested. The Post Hoc test was run for multiple comparisons. Statistically significant differences among the groups were set at p<0.05.

## Results

The positive control showed bacterial growth, while the negative control showed no bacterial growth. All bacterial strains were inhibited by all test materials. The antimicrobial activities of test materials determined by the means and standard deviation of growth inhibition zones in millimeters on all test microorganisms after 24 h are shown in Table 1. The results of 24 h incubation revealed that the antimicrobial effect of CH and CEM cement on all test micro-organisms was superior to MTA. Decreasing order of inhibition zones produced by CEM cement, CH, and MTA on all microorganisms ranged from 3.375 to 7.063, 3.375 to 6.813, and 2.250 to 5.187mm, respectively. 

The highest mean diameters of inhibition zones of bacterial growth were founded in CH and CEM cement groups, while the observed zones related to MTA group were smaller. There was no statistically significant difference between the antibacterial activity of CH and CEM cement. However, CH and CEM cement showed significantly better antibacterial effect than MTA (p<0.001).

## Discussion

In this experiment, we used ADT, which is the most widely used *in vitro *method for the evaluation of antimicrobial activity (24). This method indicates which materials are more likely to have antimicrobial activity within the root canal system *via* direct comparisons between them (25). ADT results are highly influenced by the diffusion ability of the material across the medium (26). However, the selection of the agar medium and microorganisms, control and standardization of inoculation density, incubation and reading point of the zones of inhibition are factors that affect the results of diffusion tests in an agar medium. Indeed, many different media, different methods of inoculum preparation or both have been used (27). 

The selection of test bacteria for this experiment was either true endodontic pathogens or associated with therapy-resistant cases (2). Although aerobic and facultative bacteria are usually minor constituents of primary infections, they have been found with higher frequency in cases with failed treatment (28). These bacteria can enter the root canal system before, during or after treatment and then cause secondary infections (29). An attempt was made to select representative Gram-negative / positive and cocci / bacilli bacteria that have been commonly isolated from endodontic infection.

In this study, freshly mixed cements were immediately transferred into agar plates. Because of various transitory or permanent products, material should be tested immediately after mixing and also after a period of time when it is assumed that it has reached its final chemical structure. CH and MTA are inserted into the tooth in a freshly mixed, incompletely set stage, and thus it is likely that during a period after clinical application of the material, local responses are provoked by components only partially reacted or un-reacted. After the setting period, the release of active ingredients from the materials is still possible. The difference in antibacterial patterns of various materials also may be related to the degree of setting (6).

CH shown to be appropriate for elimination of bacteria depends on ionization that releases hydroxyl ions, causing an increase in pH. A pH greater than nine may reversibly or irreversibly inactive cellular membrane enzymes of the microorganism, resulting in a loss of biological activity (30). The culture medium can influence the solubility, liberation of ions and alkalinity of CH, which are essential conditions for the antimicrobial effect (31). However our results showed effective antibacterial activity of CH which caused greater growth inhibited zones of tested bacteria than MTA and agree with those of Amorim *et al.* reported that calcium hydroxide paste formed the inhibition zones around *S. aureus, E. faecalis, P. aeruginosa, B. subtilis *and* C. albicans* strains (32). 

The antimicrobial activity of MTA was reported by Torabinejad *et al.* (5), who detected its efficiency against some facultative bacteria; however, no activity was found for *E. faecalis, S. aureus, B. subtilis *and* E. coli* or against anaerobic bacteria. Estrela *et al.* demonstrated that MTA did not reveal any antimicrobial activity against *S. aureus, E. faecalis, P. aeruginosa, B. subtilis, C. albicans* (30). In this study, the same poor result obtained with MTA. 

Our results showed effective antibacterial activity of CEM cement which is comparable with CH and significantly better than MTA group. This result could imply that CEM cement contains more potent antibacterial inhibitors than MTA. Alkaline earth metal oxides and hydroxides (e.g. calcium oxide and calcium hydroxide), calcium phosphate, and calcium silicate are the important constituents of CEM cement. During mixing with its liquid and after that, hydration reactions take place that result in the production of CH, largely because of the reactions involving calcium silicates, calcium phosphate, and calcium oxide in addition to presence of CH alone. When CEM cement transfers to agar plates and contacts to the medium, CH dissociated into calcium and hydroxyl ions increasing the pH and calcium concentrations. These mechanisms may be partially explained favored antibacterial activity of this material. An alternative explanation is that the antibacterial components of CEM cement have better diffusion properties.

## Conclusion

Under the conditions of this *in vitro* study it was concluded that the favored results of CEM cement and CH in comparison with MTA, indicate potentiality of CEM cement usage as antibacterial agent. However, it is necessary to investigate other properties of this novel endodontic material.
